# Luminescent properties of Eu^3+^-activated Gd_2_ZnTiO_6_ double perovskite red-emitting phosphors for white light-emitting diodes and field emission displays†

**DOI:** 10.1039/c8ra00700d

**Published:** 2018-03-21

**Authors:** Soo Hyun Lee, Youngjin Cha, Hyosung Kim, Seungmoo Lee, Jae Su Yu

**Affiliations:** Department of Electronic Engineering, Kyung Hee University Yongin-si Gyeonggi-do 17104 Republic of Korea jsyu@khu.ac.kr +82 31 204 8115 +82 31 201 3820

## Abstract

We synthesized a series of double perovskite Eu^3+^-activated Gd_2_ZnTiO_6_ red-emitting phosphors by a solid-state reaction route and analyzed their morphology, crystallinity, luminescent properties, and thermal stability. Under 270 nm of excitation, the prominent emission peak of the phosphors was found to be located in the red region with the central wavelength of 613 nm corresponding to the intra-4f transition of Eu^3+^ ions from the ^5^D_0_ to ^7^F_2_ level. The optimum concentration of the activator was determined to be 7 mol%. The studied phosphors also exhibited good thermal stability with the activation energy of 0.233 eV. The white color emitted from the ultraviolet (UV) light-emitting diode device which was coated by commercial blue-/green-emitting phosphors and Gd_1.86_ZnTiO_6_:0.14Eu^3+^ phosphors exhibited a high color rendering index of 82.9. Furthermore, the cathodoluminescence performance of the resultant phosphors was also investigated in detail. These characteristics of Gd_2−2*x*_ZnTiO_6_:2*x*Eu^3+^ phosphors make them potential candidates for UV-based white light-emitting diodes and field emission displays.

## Abstract 2

We synthesized a series of double perovskite Eu^3+^-activated Gd_2_ZnTiO_6_ red-emitting phosphors by a solid-state reaction method and analyzed their morphology, crystallinity, luminescent properties, and thermal stability.

## Graphical abstract

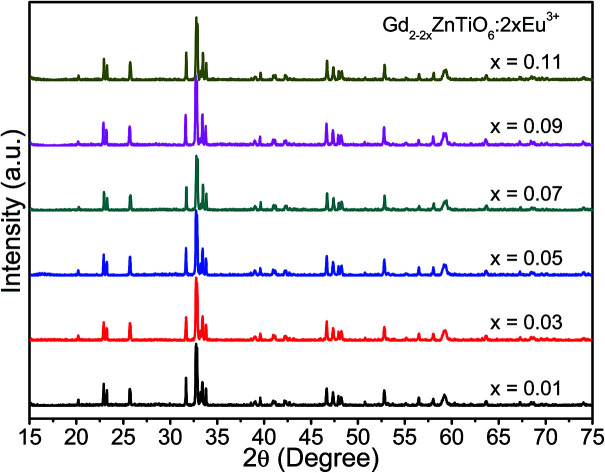

## 1. Introduction

Currently, phosphor-converted white light-emitting diodes (WLEDs) have been extensively adopted in various solid-state lighting applications due to their superior characteristics such as high efficiency, energy saving, long lifetime, and environmental friendliness compared to the conventional incandescent and fluorescent light bulbs.^1–14^ Commonly, the strategy to design commercial WLEDs is the combination of InGaN blue LEDs and Y_3_Al_5_O_12_:Ce^3+^ yellow-emitting phosphors. However, the white light obtained from this configuration possesses a low color rendering index (CRI < 80) due to the lack of red component, leading to the restriction of their use in practical applications.^3–5^ To overcome this issue, new designs for white colors emitted from blends of tricolor (red, green, and blue) phosphors excited by ultraviolet (UV) chips have been developed.^6–14^ Since these tricolor-phosphors-based WLEDs exhibit excellent CRI (>80) and high conversion efficiency, the exploration of luminescent materials is a big challenge. In particular, although several highly efficient red-emitting phosphors have been investigated, there are still some technical limitations such as complexity in synthetic processes, harsh environment, and high cost. Therefore, the development of novel and high-performance red-emitting phosphors pumped by UV light is significantly demanded.

Up to date, lots of inorganic materials, such as molybdates, tungstates, titanates, oxides, and silicates, have been successfully developed for luminescent host materials.^15–20^ Especially, doubly ordered perovskites, with the general formula of AA′BB′O_6_, A_2_BB′O_6_, and AB_1/3_B′_2/3_O_3_, have attracted great attention due to their stable crystalline structure and high thermal stability which make them suitable for luminescent host materials.^21–25^ Among them, Gd_2_ZnTiO_6_ has been recently reminded due to the discovery of its monoclinic structure.^26–28^ Previously, the announced crystal structure of Gd_2_ZnTiO_6_ was only orthorhombic. Das *et al.* synthesized the monoclinically distorted perovskites (*i.e.*, A_2_ZnTiO_6_ (A = Pr, Gd)) *via* the solid-state reaction method.^26^ They refined the lattice parameters and found the completed order of B site cations (Zn and Ti) in Gd_2_ZnTiO_6_. Chen *et al.* reported that the Mn^4+^ ions were expected to occupy the sites of Ti^4+^ ions in the Gd_2_ZnTiO_6_ host lattice and the resultant compounds can emit bright red emission.^27^ Furthermore, it was also demonstrated that the Gd_2_ZnTiO_6_:Mn^4+^/Er^3+^ phosphors were promising luminescent materials for simultaneous upconversion and downconversion emissions.^28^ Although many admirable results have been achieved in the rare-earth doped Gd_2_ZnTiO_6_, more efforts are still needed to further investigate the luminescent properties of Gd_2_ZnTiO_6_.

As is known, the Eu^3+^ ions, as a part of the rare-earth ions, have been widely used to obtain red emission. Generally, Eu^3+^ ions show two typical emissions, namely, yellow emission at around 591 nm (^5^D_0_ → ^7^F_1_) and red emission at about 613 nm (^5^D_0_ → ^7^F_2_).^29–31^ On the basis of previous literatures, it is evident that the dominant emission of Eu^3+^ ions in the double perovskite structure materials was decided by doping sites and lattice structure of host material.^25,32^ When Eu^3+^ ions substitute B sites (center of octahedron) and A sites with centrosymmetry (*e.g.,* pseudo-cubic), the ^5^D_0_ → ^7^F_1_ transition becomes dominant, while the ^5^D_0_ → ^7^F_2_ transition is dominant when the Eu^3+^ ions occupy the A sites with non-centrosymmetry (*e.g.,* monoclinic). Thus, it could be expected that vivid red emission is generated from Eu^3+^-activated monoclinic Gd_2_ZnTiO_6_ (Gd_2_ZnTiO_6_:Eu^3+^) phosphors. Furthermore, the Eu^3+^ ions can be selected as a proper activator in Gd_2_ZnTiO_6_ lattice because the ionic radius of Eu^3+^ (1.29 Å, coordinate number (CN) = 12) is almost similar to that of Gd^3+^ (1.27 Å, CN = 12), leading to no significant crystalline and phase distortions after doping. In this work, thus, the Gd_2_ZnTiO_6_:Eu^3+^ red-emitting phosphors were prepared *via* a high-temperature solid-state reaction method. The morphological properties were observed by using a field-emission scanning electron microscope (FE-SEM). The phase and crystalline properties were examined by using an X-ray diffractometer and a transmission electron microscope (TEM). Their luminescent properties and temperature dependency were also investigated. The optimum products were applied in WLED applications. For multifunctional purpose, their cathodoluminescence (CL) properties were investigated under different operating conditions for field emission display (FED) systems.

## 2. Experimental details

The Gd_2−2*x*_ZnTiO_6_:2*x*Eu^3+^ red-emitting phosphors with various Eu^3+^ concentrations (*x* = 0.01, 0.03, 0.05, 0.07, 0.09, and 0.11) were synthesized *via* a high-temperature solid-state reaction technique. The gadolinium(iii) oxide (Gd_2_O_3_, 99.9%), zinc oxide (ZnO, ≥99.0%), titanium(iv) oxide (TiO_2_, ≥99%), and europium(iii) oxide (Eu_2_O_3_, 99.5%) were purchased from Sigma Aldrich Co. and used as precursors with no further purification. The raw materials with stoichiometric amounts were mixed and grinded together in an agate mortar for 10 min to obtain a homogeneous composition and then they were successively placed into alumina crucibles in a muffle furnace. Under an atmospheric environment, all the as-prepared samples were calcined with a two-step temperature profile. In the first step, the temperature was linearly increased up to 900 °C with a ratio of 5 °C min^−1^ and kept for 8 h. Next, it reached to 1200 °C with a ratio of 5 °C min^−1^ and maintained for 6 h. After the process, the final products of Gd_2−2*x*_ZnTiO_6_:2*x*Eu^3+^ phosphors were naturally cooled down to room temperature.

The morphological characteristics and elemental mapping of Gd_2−2*x*_ZnTiO_6_:2*x*Eu^3+^ phosphors were observed by employing a FE-SEM (LEO SUPRA 55, Carl Zeiss) equipped with the energy dispersive X-ray (EDX) spectroscopy system. The crystalline properties of Gd_2−2*x*_ZnTiO_6_:2*x*Eu^3+^ phosphors were investigated by using a X-ray diffractometer (M18XHF-SRA, Mac Science) with Cu Kα (*λ* = 1.5406 Å) radiation and a TEM (JEM-2100F, JEOL). The room-temperature photoluminescence (PL) excitation (PLE) and PL emission spectra of Gd_2−2*x*_ZnTiO_6_:2*x*Eu^3+^ phosphors were examined by utilizing a spectrofluorometer (Scinco FluroMateFS-2) and the temperature-dependent PL emission spectra were measured in the temperature range of 303–483 K with an interval of 20 K using a temperature controlled stage (NOVAST540). The fluorescence spectrophotometer (Photon Technology International fluorimeter) attached with a Xe flash lamp of 25 W power was used to measure the decay curve. The absorption spectrum of the optimum phosphor was recorded by using a V-670 (JASCO) UV-vis spectrophotometer. Their CL properties were also taken by using a Gatan (UK) MonoCL3 system attached with the SEM (Hitachi S-4300 SE).

## 3. Results and discussion

**Fig. 1:**
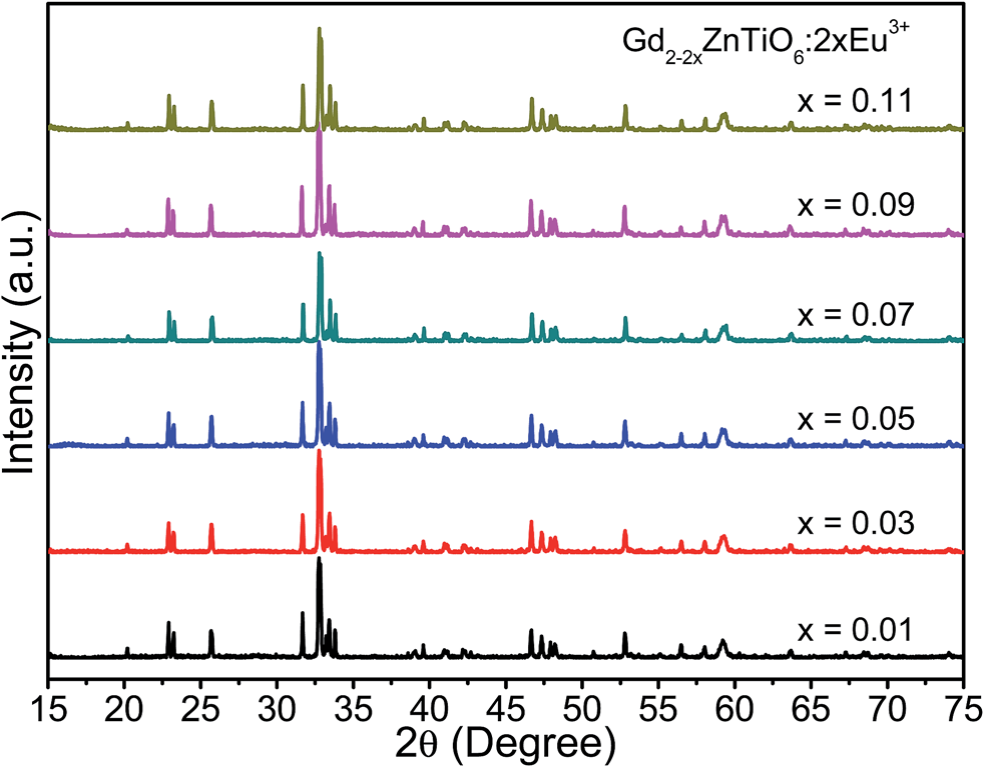
XRD patterns of the Gd_2−2*x*_ZnTiO_6_:2*x*Eu^3+^ (*x* = 0.01, 0.03, 0.05, 0.07, 0.09, and 0.11) phosphors in the 2*θ* range of 15–75°.

Fig. 1 shows the X-ray diffraction (XRD) patterns of the Gd_2−2*x*_ZnTiO_6_:2*x*Eu^3+^ (*x* = 0.01, 0.03, 0.05, 0.07, 0.09, and 0.11) phosphors in the 2*θ* range of 15–75°. Since the monoclinic Gd_2_ZnTiO_6_ has been recently reported, there is no standard information yet. The refinement parameters of monoclinic Gd_2_ZnTiO_6_ were calculated by Das *et al.* and Chen *et al.* using a FullProf program, which were used as the ref. 26 and 27. For all the Gd_2−2*x*_ZnTiO_6_:2*x*Eu^3+^ phosphors, as shown in Fig. 1, the diffraction peaks were consistent to its monoclinic phase with a space group of *P*2_1_/*n* (no. 14). No other peaks were observed, indicating a pure phase of Gd_2_ZnTiO_6_. With the introduction of Eu^3+^ ions, no peaks were significantly shifted, implying the successful doping of Eu^3+^ ions into the Gd_2_ZnTiO_6_ host lattice, which was also able to be confirmed from a tolerance factor (*τ*). The *τ* of double perovskite group (A_2_BB′O_6_) can be calculated by using the equation:^33^1
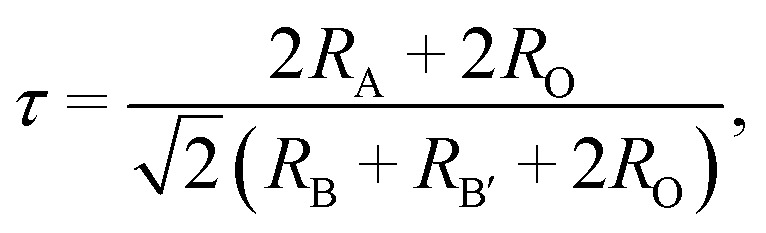
where *R*_A_, *R*_B_, *R*_B′_, and *R*_O_ is the ionic radius of A-cation, zinc, titanium, and oxygen, respectively. It is known that the deviation of *τ* from unity indicates the distortion in structures. For the Gd_1.86_ZnTiO_6_:0.14Eu^3+^, herein, the values of *R*_A_, *R*_B_, *R*_B′_, and *R*_O_ were used to be 1.27 Å (Gd^3+^, CN = 12), 0.74 Å (Zn^2+^, CN = 6), 0.605 Å (Ti^4+^, CN = 6), 1.35 Å (O^2−^, CN = 4), and 1.29 Å (Eu^3+^, CN = 12), respectively. As a consequence, the value of *τ* was calculated to be 0.92. This *τ* value, close to unity, represents the less distortion in monoclinic structure due to the small difference in ionic radius between Gd^3+^ and Eu^3+^ ions and lowered symmetry.^33,34^

**Fig. 2:**
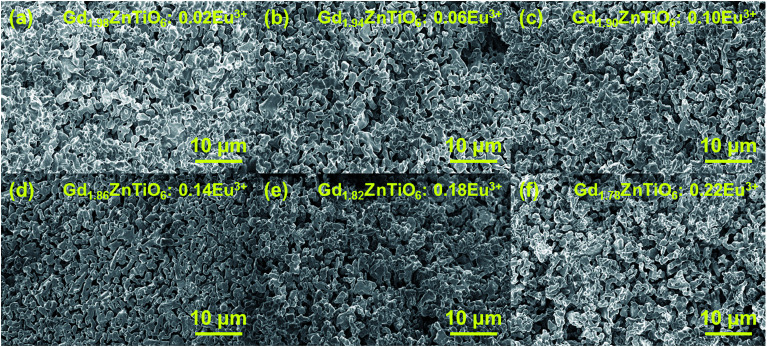
Surface morphologies of the Gd_2−2*x*_ZnTiO_6_:2*x*Eu^3+^ (*x* = (a) 0.01, (b) 0.03, (c) 0.05, (d) 0.07, (e) 0.09, and (f) 0.11) phosphors.

Fig. 2 shows the surface morphologies of the Gd_2−2*x*_ZnTiO_6_:2*x*Eu^3+^ (*x* = 0.01, 0.03, 0.05, 0.07, 0.09, and 0.11) phosphors. It is found that the morphologies were independent of doping concentration. The particles exhibited the non-uniform shapes with an average size of 2 μm and large aggregations. This was attributed to the high-temperature treatment in solid-state reaction technique.

**Fig. 3:**
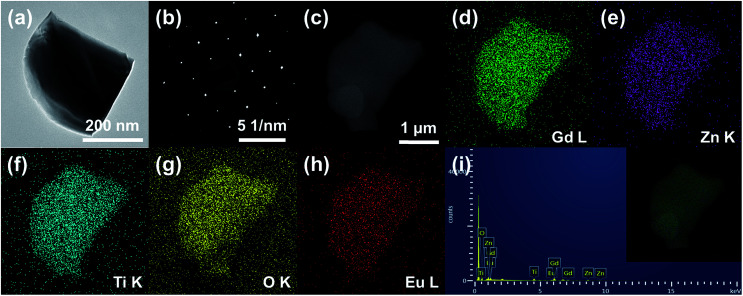
(a) TEM image, (b) SAED pattern, (c) SEM image, (d–h) elemental mapping images, and (i) EDX spectrum of the Gd_1.86_ZnTiO_6_:0.14Eu^3+^ phosphor.

Fig. 3 shows the (a) TEM image, (b) selected area electron diffraction (SAED) pattern, (c) SEM image, (d–h) elemental mapping images, and (i) EDX spectrum of the Gd_1.86_ZnTiO_6_:0.14Eu^3+^ phosphor. The aligned dot patterns were confirmed in the SAED pattern, which obviously indicates the high-quality single crystalline nature of particle. From the elemental mapping results, the uniform contribution of constituent elements (Gd, Zn, Ti, O, and Eu) was also observed. The qualitative information of phosphor was represented in its EDX spectrum. Apart from Gd, Zn, Ti, O, and Eu, no other peaks were detected, further confirming the completion of Eu^3+^-activated Gd_2_ZnTiO_6_ phosphors.

**Fig. 4:**
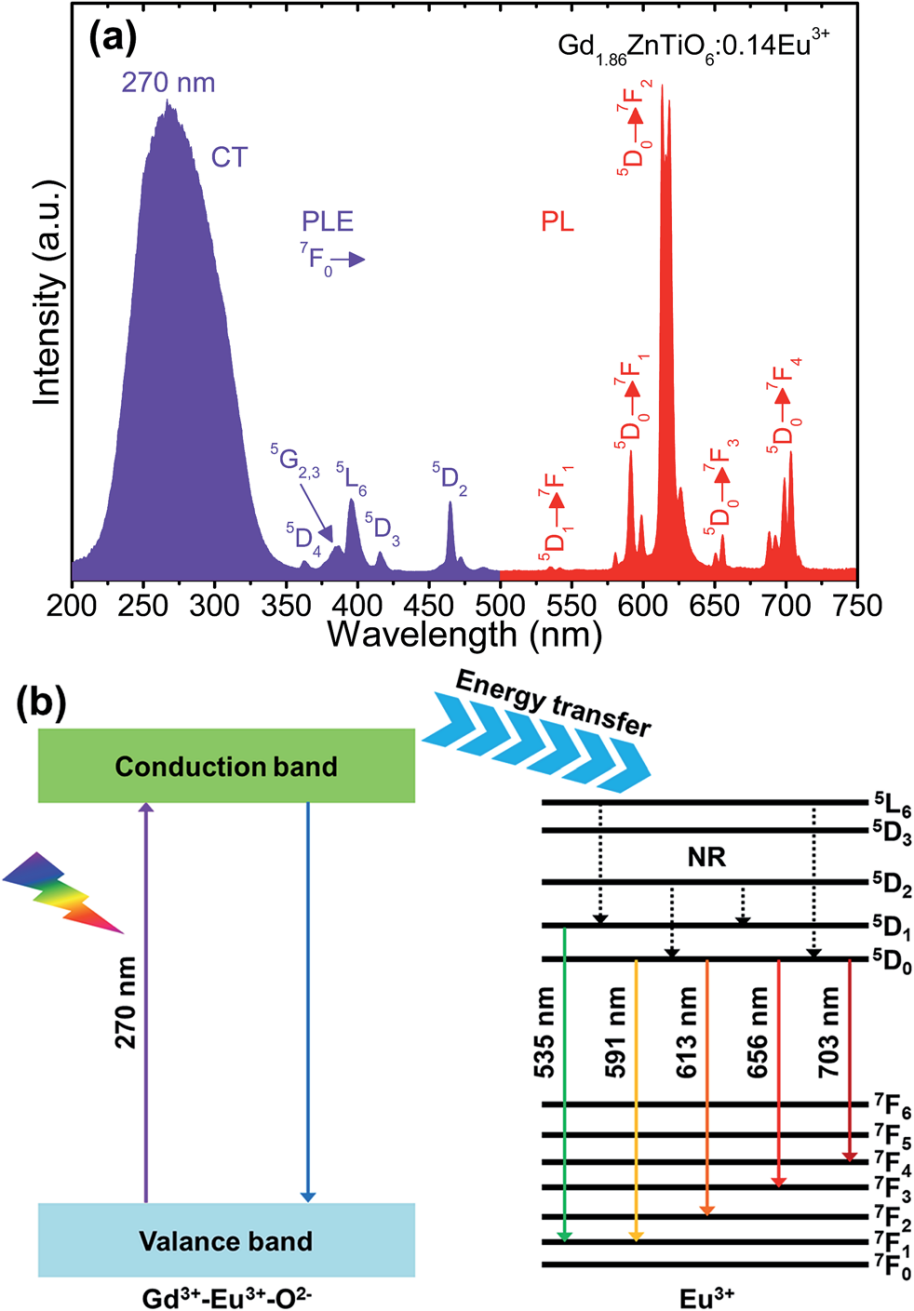
(a) PLE and PL emission spectra of the Gd_1.86_ZnTiO_6_:0.14Eu^3+^ phosphor and (b) energy level diagram of the Gd_2_ZnTiO_6_ host material and Eu^3+^ ions.

Fig. 4(a) shows the typical PLE and PL emission spectra of the Gd_1.86_ZnTiO_6_:0.14Eu^3+^ phosphor. The PLE spectrum was recorded in the wavelength range of 200–500 nm while the PL emission spectrum was measured at the wavelengths of 500–750 nm. As for the PLE spectrum (*λ*_em_ = 613 nm), one broadband and several narrow peaks were observed. The broadband with a center wavelength of 270 nm was attributed to the electron transfer from the 2p orbit of O^2−^ ions to the 4f orbit of Gd^3+^ and Eu^3+^ ions, *i.e.*, as-called the charge transfer (CT) band. The other peaks at 362, 377, 384, 396, 416, and 464 nm belonged to the transitions of ^7^F_0_ → ^5^D_4_, ^7^F_0_ → ^5^G_2_, ^7^F_0_ → ^5^G_3_, ^7^F_0_ → ^5^L_6_, ^7^F_0_ → ^5^D_3_, and ^7^F_0_ → ^5^D_2_ of Eu^3+^ ions, respectively.^22,23^ From the result, the absorption in CT band was much higher than that of Eu^3+^ ions, suggesting that the UV light can act as the light source for the studied samples. The PL emission spectrum was measured under the illumination at 270 nm. The typical peaks of Eu^3+^ ions were detected at 535 nm (^5^D_1_ → ^7^F_1_), 591 nm (^5^D_0_ → ^7^F_1_), 613 nm (^5^D_0_ → ^7^F_2_), 656 nm (^5^D_0_ → ^7^F_3_), and 703 nm (^5^D_0_ → ^7^F_4_).^35–38^ The PL emission spectrum at 396 nm also exhibited the same characteristics, leading to that the Gd_2−2*x*_ZnTiO_6_:2*x*Eu^3+^ phosphors are a promising candidate for practical applications with near-UV (NUV) LEDs (Fig. S1†). In general, the ^5^D_0_ → ^7^F_1_ transition is allowed by the magnetic dipole, while the ^5^D_0_ → ^7^F_2_ transition is allowed by the forced electrical dipole. The magnetic dipole is not sensitive to the local environment as well as the crystal field, while the forced electrical dipole is.^25,35,39^ Clearly, in present work, the emission intensity at 613 nm was obviously larger than that at 591 nm. Thus, this phenomenon implies that the Eu^3+^ ions were located onto the sites without inversion symmetry in host material.

For better comprehension of luminescent mechanism of Gd_2−2*x*_ZnTiO_6_:2*x*Eu^3+^ phosphors, the energy level diagram of the Gd_2_ZnTiO_6_ host material and Eu^3+^ ions was simply demonstrated in Fig. 4(b). When the powders are exposed to UV light of which range corresponds to the CT band, especially at 270 nm, electron–hole pairs are generated in the Gd_2_ZnTiO_6_ host material. The excited electrons move from the valance to conduction band. Next, the energy is transferred to the ^5^L_6_ level of Eu^3+^ ions and then non-radiatively transited downward to the ^5^D_1_ and ^5^D_0_ levels. At last, the radiative transitions to the ground states (^7^F_*J*_, *J* = 1, 2, 3, and 4) were performed, leading to the formation of characteristic emissions of Eu^3+^ ions.

**Fig. 5:**
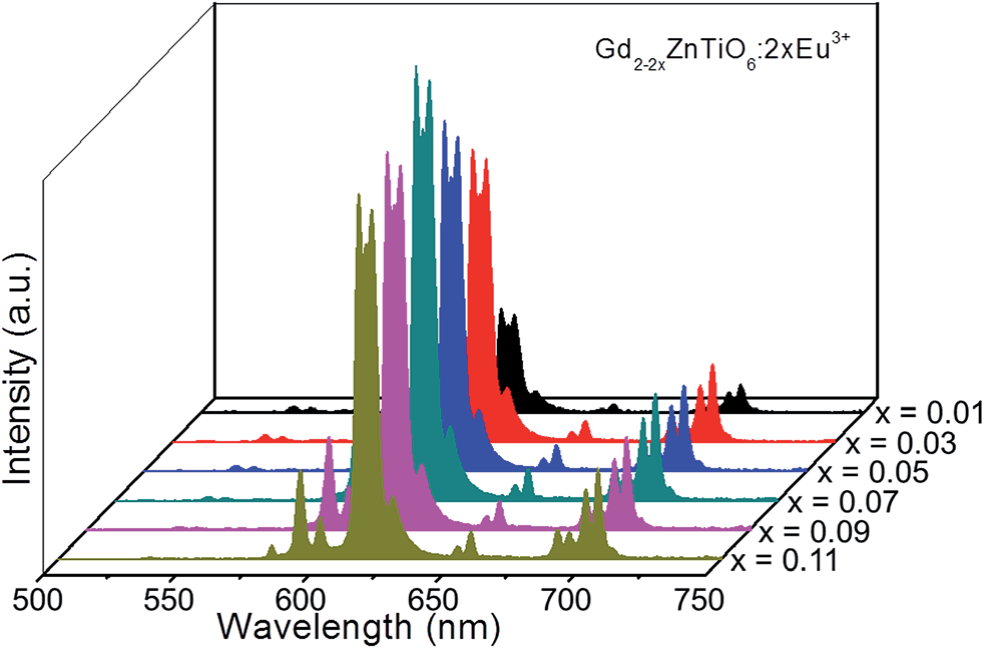
PL emission spectra of the Gd_2−2*x*_ZnTiO_6_:2*x*Eu^3+^ (*x* = 0.01, 0.03, 0.05, 0.07, 0.09, and 0.11) phosphors.

Fig. 5 shows the PL emission spectra of the Gd_2−2*x*_ZnTiO_6_:2*x*Eu^3+^ (*x* = 0.01, 0.03, 0.05, 0.07, 0.09, and 0.11) phosphors. Under different doping concentrations, the spectral shapes and peak positions were almost consistent. The PL emission intensity at 613 nm was dramatically increased along the doping concentration and its highest value was achieved at *x* = 0.07. Beyond the optimum concentration (*x* > 0.07), the emission intensity was gradually decreased. Obviously, the distance between Eu^3+^ ions becomes smaller at higher doping concentration. When the Eu^3+^ ions are much closer one another, the cross relaxation with NR energy transfer occurs, leading to the lower efficiency.^40,41^ This concentration quenching can be related to either the exchange interaction or multipole interaction.^42,43^ Herein, the critical distance (*R*_c_) could be roughly used to state the dominant mechanism for concentration quenching effect. For *R*_c_ < 5 Å, it is considered that the exchange interaction is dominant in the concentration quenching mechanism, otherwise the multipole interaction takes the domination. The *R*_c_ is evaluated by the equation proposed by Blasse and Bril:^44,45^2
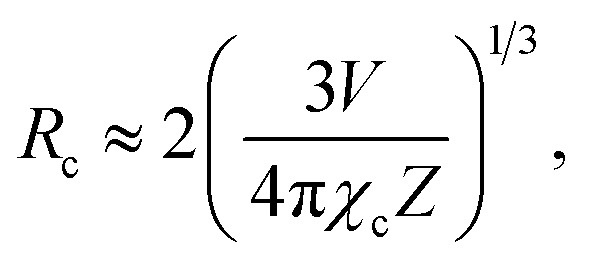
where *V* is the volume of the unit cell, *Z* is the number of cations in the unit cell, and *χ*_c_ is the critical doping concentration of activator. Herein, the values of *V*, *Z*, and *χ*_c_ were used to be 233.315 Å^3^, 2, and 0.07, respectively, resulting in the value of *R*_c_ as 14.71 Å. Since the *R*_c_ of Gd_1.86_ZnTiO_6_:0.14Eu^3+^ was larger than 5 Å, it is reasonable for us to consider that the multipole interaction contributes to the concentration quenching effect of Eu^3+^ ions in the Gd_2_ZnTiO_6_ host material.

**Fig. 6:**
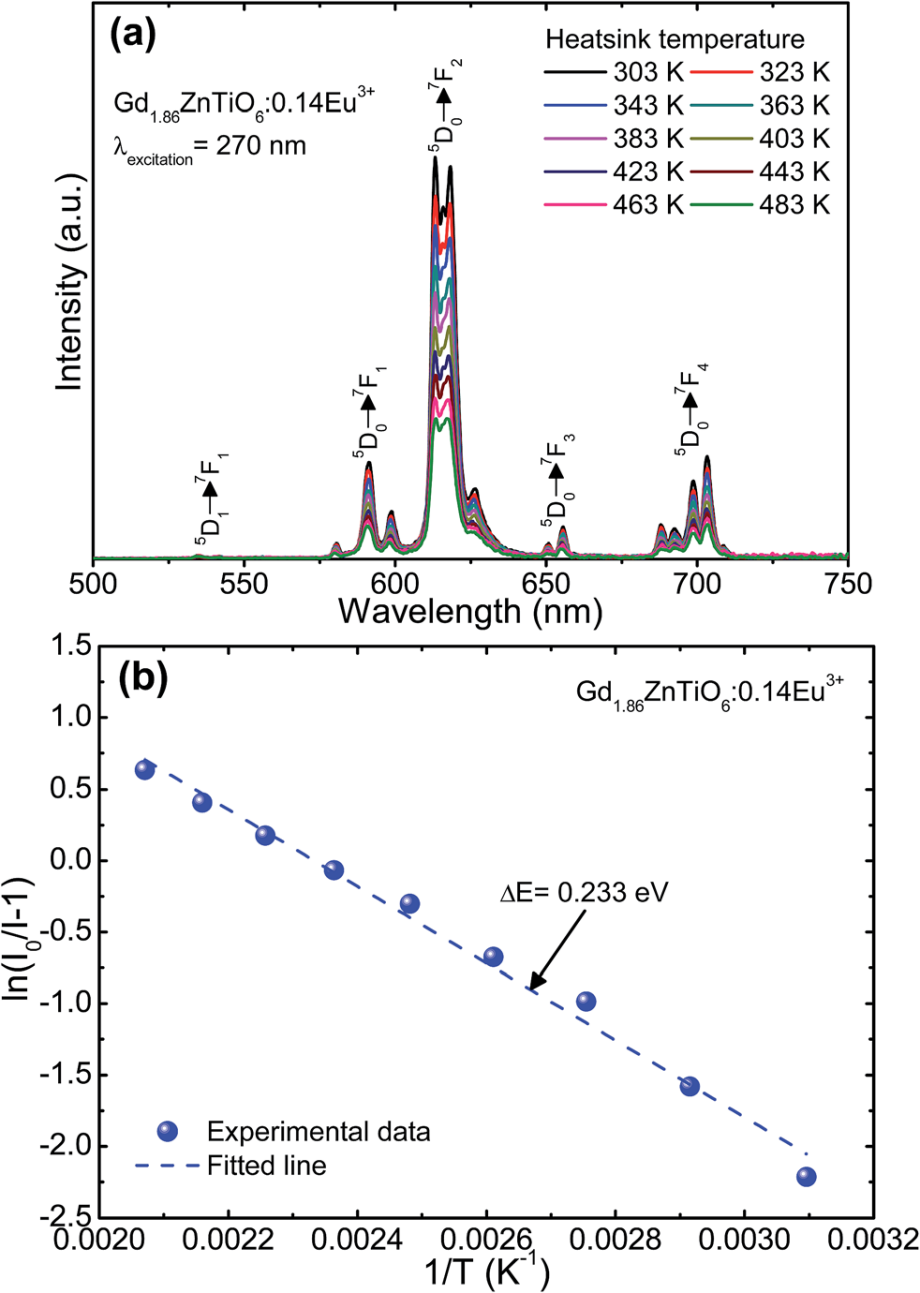
(a) Temperature-dependent PL emission spectra in the temperature range of 303–483 K with an interval of 20 K and (b) plot of ln(*I*_0_/*I* − 1) *versus* 1/*T* of the Gd_1.86_ZnTiO_6_:0.14Eu^3+^ phosphor.

To explore the potential application of the as-fabricated phosphors for WLEDs, the thermal stability was evaluated. Fig. 6 shows the (a) temperature-dependent PL emission spectra in the temperature range of 303–483 K with an interval of 20 K and (b) plot of ln(*I*_0_/*I* − 1) *versus* 1/*T* of the Gd_1.86_ZnTiO_6_:0.14Eu^3+^ phosphor. The PL emission intensity was decreased as the temperature was elevated, as presented in Fig. 6(a). The thermal stability of phosphors can be evaluated by the quenching temperature at which the half of initial PL intensity is observed. For the Gd_1.86_ZnTiO_6_:0.14Eu^3+^ phosphor, the PL emission intensity at 423 K with respect to that at 303 K was decreased by 51.7% due to the thermal quenching effect. For further understanding the involved thermal quenching phenomenon, the activation energy (Δ*E*) was calculated utilizing the Arrhenius equation:^46^3
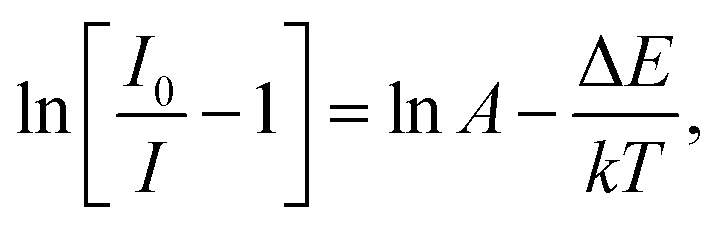
where *T* is the temperature, *I*_0_ is the initial emission intensity, *I* is the emission intensity at *T*, *A* is the constant, and *k* is the Boltzmann constant. From the plot of ln(*I*_0_/*I* − 1) *versus* 1/*T* (see Fig. 6(b)), the Δ*E* was extracted to be 0.233 eV. Evidently, the obtained quenching temperature and Δ*E* were comparable with the pervious developed Eu^3+^-activated red-emitting phosphors, such as Na_0.5_Gd_0.5_MoO_4_:Eu^3+^ (443 K and 0.235 eV), Sr_3_Sn_2_O_7_:Eu^3+^ (434 K and 0.263 eV), Na_2_Gd(PO_4_)(MoO_4_):Eu^3+^ (463 K and 0.22 eV), and Ca_2_La_8_(SiO_4_)_6_O_2_:Eu^3+^ (463 K and 0.207 eV),^47–50^ revealing that the Eu^3+^-activated Gd_2_ZnTiO_6_ phosphors possess good thermal stability and are promising candidates for WLEDs as red-emitting phosphors.

**Fig. 7:**
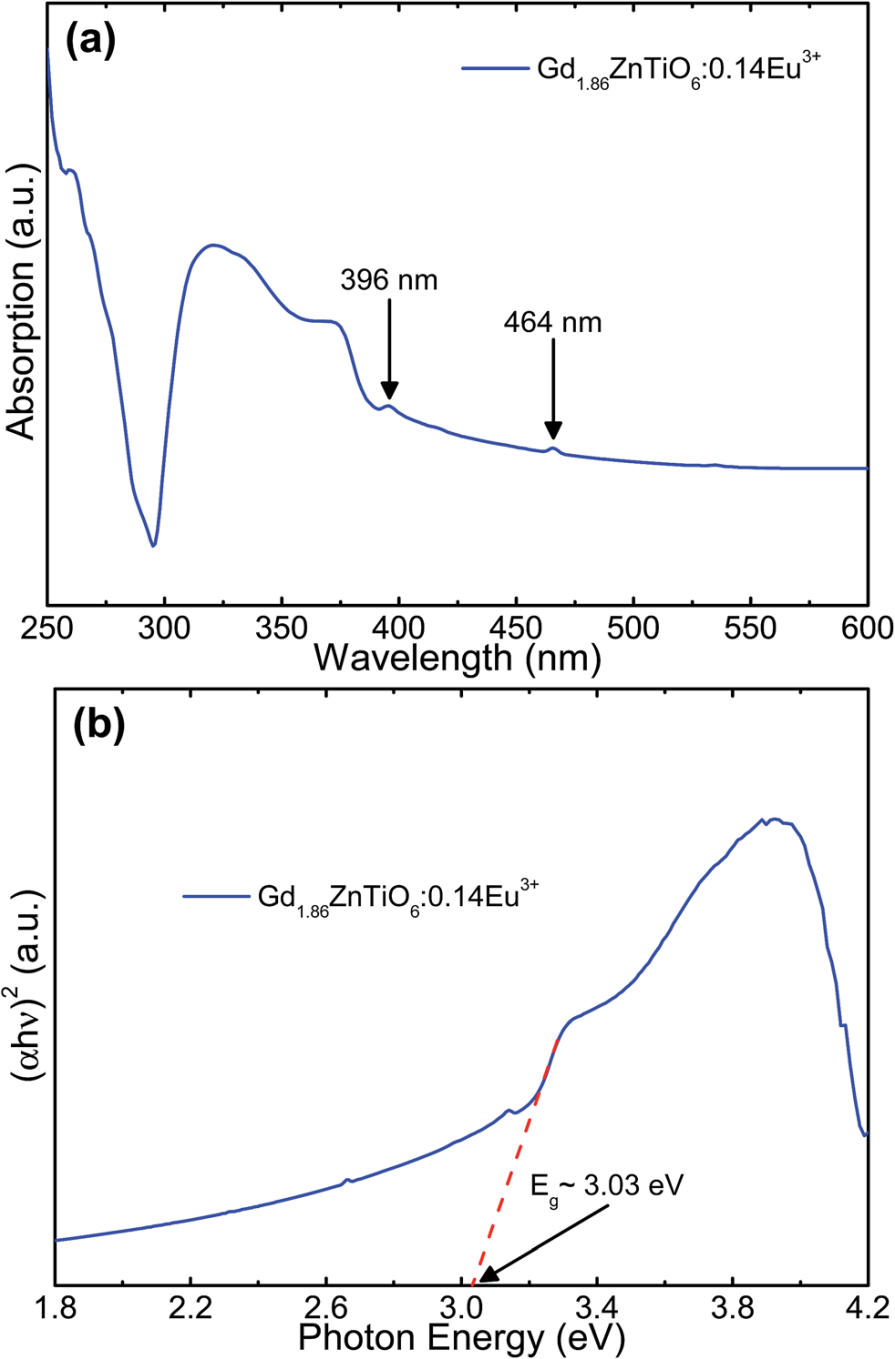
(a) Absorption spectrum and (b) plot of (*αhν*)^2^*versus* photon energy of the Gd_1.86_ZnTiO_6_:0.14Eu^3+^ phosphor.

Fig. 7 shows the (a) absorption spectrum and (b) plot of (*αhν*)^2^*versus* photon energy of the Gd_1.86_ZnTiO_6_:0.14Eu^3+^ phosphor. As shown in Fig. 7(a), the strong and wide absorption region was found in the wavelength range below 300 nm, which was assigned to the CT band, while these narrow peaks at 396 and 464 nm belonged to the Eu^3+^ ions. This behavior is in good agreement with the PLE spectrum as shown in Fig. 4(a). Furthermore, the optical band gap, *E*_g_, can be roughly evaluated by following the Wood and Tauc relation:^51^

4 (*ahν*)^2^ ≈ *A*(*hν* − *E*_g_),

where *α* is the absorption coefficient, *hν* is the photon energy, and *A* is the constant. From the curve, the *E*_g_ of Gd_1.86_ZnTiO_6_:0.14Eu^3+^ phosphor was determined by the extrapolation to of the linear portion of the plot of (*αhν*)^2^ = 0 and its value was represented to be ∼3.07 eV.

**Fig. 8:**
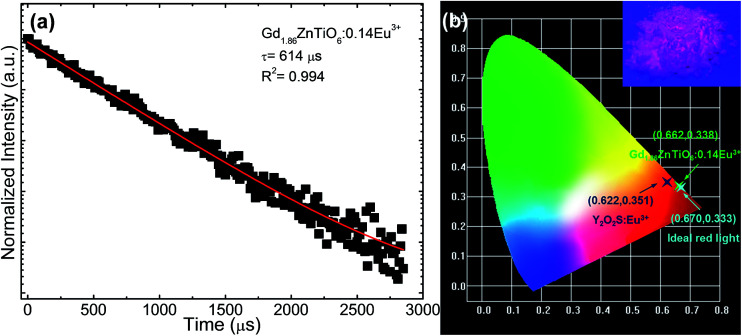
(a) Luminescence decay curve and (b) CIE chromaticity coordinate of the Gd_1.86_ZnTiO_6_:0.14Eu^3+^ phosphor. The vivid and intense red color was clearly observed from the powders by naked-eyes as shown in the photograph of (b).

Fig. 8(a) shows the luminescence decay curve of the Gd_1.86_ZnTiO_6_:0.14Eu^3+^ phosphor. The decay curve for the ^5^D_0_ → ^7^F_2_ transition (613 nm) of Eu^3+^ ions was fitted using a single exponential function which is expressed as:

5 *I* = *I*_0_ exp(−*t*/*τ*) + *A*,

where *t* is the time, *I* and *I*_0_ are the luminescence intensities at *t* and *t* = 0, *τ* is the lifetime, and *A* is the constant. According to the fitting result, the lifetime was demonstrated to be 614 μs. The Commission International de I'Eclairage (CIE) chromaticity coordinates of the Gd_1.86_ZnTiO_6_:0.14Eu^3+^ phosphor are represented in Fig. 8(b). The color coordinates were determined from the PL spectra under the excitation at 270 nm as shown in Fig. 4, indicating its value to be (*x* = 0.662, *y* = 0.338) located in the edge of the red region. In comparison, the estimated CIE coordinate of the studied samples was close to that of the ideal red light (0.670, 0.333) and much better than that of the commercial Y_2_O_2_S:Eu^2+^ red-emitting phosphors (0.622, 0.351), suggesting that the prepared materials exhibited superior chromatic properties. To further comprehend the chromatic performance of the Eu^3+^-activated Gd_2_ZnTiO_6_ phosphors, the color purity was evaluated using the equation of:^49,52^6
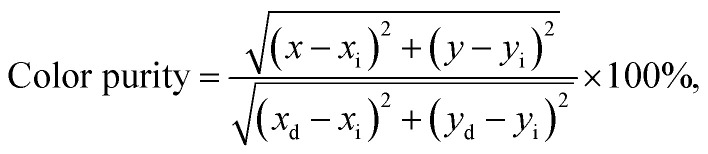
where (*x*_i_, *y*_i_) is the CIE chromaticity coordinate of the white illumination and (*x*_d_, *y*_d_) is the CIE chromaticity coordinate of the dominant wavelength. Here, the (*x*_i_, *y*_i_) was of (0.310, 0.316) and the (*x*_d_, *y*_d_) was of (0.674, 0.325). As a consequence, the color purity of the final product was determined to be as high as 96.8% which was much higher than the previous reported works, such as SrMoO_4_:Eu^3+^ (85.8%), Gd_2_MoO_6_:Eu^3+^ (95.8%), and CaW_0.4_Mo_0.6_O_4_:Eu^3+^ (93.8%),^35,53,54^ further implying the Eu^3+^-activated Gd_2_ZnTiO_6_ phosphors had splendid chromatic behaviors. Under the irradiation of NUV light, the prepared samples emitted vivid and intense red emission that can be seen by naked-eyes, as shown in the inset of Fig. 8(b).

**Fig. 9:**
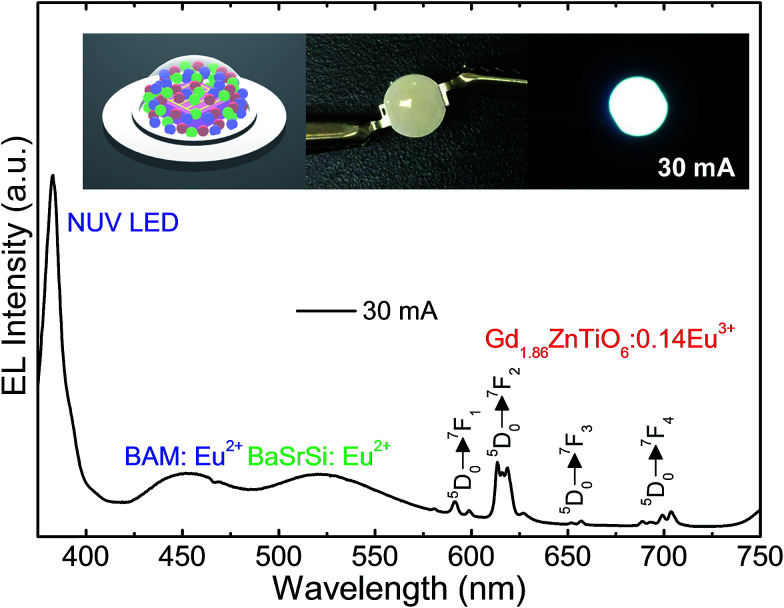
EL spectrum of the WLED fabricated by the combination of UV LED (*λ* ∼ 385 nm), commercial blue-emitting and green-emitting phosphors, and Gd_1.86_ZnTiO_6_:0.14Eu^3+^ red-emitting phosphor at the injection current of 30 mA under continuous-wave mode. The schematic illustration of WLED and its photographs before/after the current injection were demonstrated in the inset.

For the purpose of exploring the potential application of the studied samples for indoor illumination, an NUV chip-based WLED device, which consisted of combination of UV LED (*λ* ∼ 385 nm), commercial blue-emitting (BAM:Eu^2+^, *i.e.*, BaMgAl_10_O_17_:Eu^2+^) and green-emitting (BaSrSi:Eu^2+^, *i.e.*, (Ba, Sr)_2_SiO_4_:Eu^2+^) phosphors, and Gd_1.86_ZnTiO_6_:0.14Eu^3+^ red-emitting phosphor, was prepared. Fig. 9 shows the EL spectrum of the WLED device at the injection current of 30 mA under continuous-wave mode. The schematic illustration of the as-fabricated WLED was demonstrated in the inset of Fig. 9. Under the 30 mA of forward bias current, the clearly distinguishable tricolor bands and peaks were observed. In particular, all the characteristic peaks of Eu^3+^ ions were found without any distortion compared to the PL spectra of Gd_1.86_ZnTiO_6_:0.14Eu^3+^ phosphor (see Fig. 4). Under the forward bias current of 30 mA, the fabricated LED device emitted bright white light with high CRI value of 82.9. The photographs of WLED before/after the current injection are also shown in the inset of Fig. 9. This result strongly implies that the Eu^3+^-activated Gd_2_ZnTiO_6_ red-emitting phosphors were a promising candidate for practical WLED industry.

**Fig. 10:**
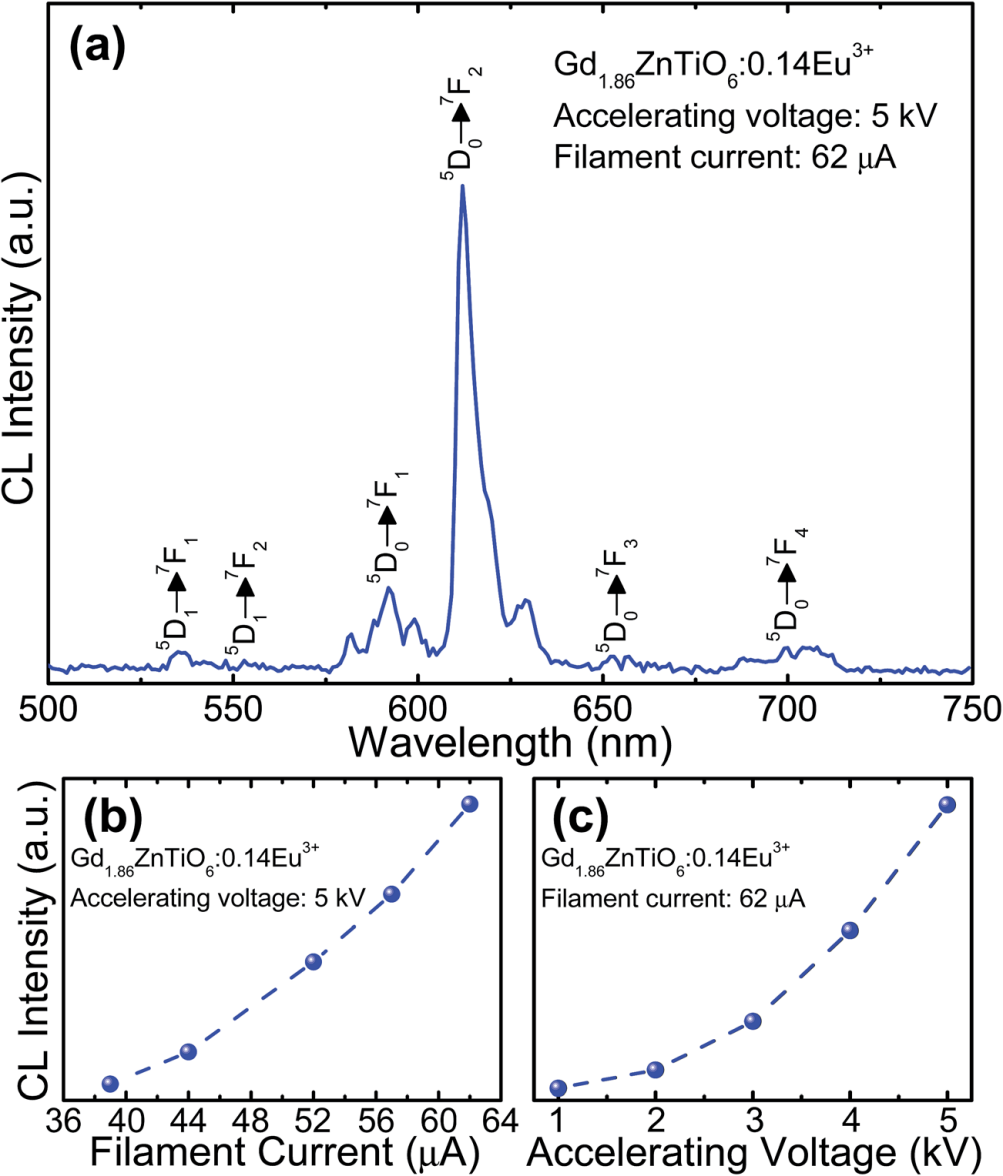
(a) CL spectrum of the powder under the filament current of 62 μA at the accelerating voltage of 5 kV, and CL intensities (b) under different filament currents when the accelerating voltage was fixed at 5 kV and (c) under different accelerating voltages when the filament current was fixed at 62 μA.

For the development of novel and efficient red phosphors in FED application, the CL properties of the prepared phosphors were systematically explored. Fig. 10 shows the (a) CL spectrum of the powder under the filament current of 62 μA at the accelerating voltage of 5 kV, and CL intensities (b) under different filament currents when the accelerating voltage was fixed at 5 kV and (c) under different accelerating voltages when the filament current was fixed at 62 μA. As shown in Fig. 10(a), the studied samples exhibited the characteristic emissions of Eu^3+^ ions and the emission originating from the ^5^D_0_ → ^7^F_2_ transition was dominated in the CL spectrum, which coincided well with the PL spectrum. Furthermore, according to the Fig. 10(b) and (c), it is evident that the CL intensity was continuously and gradually increased by increasing the filament current and accelerating voltage without exhibiting any significant saturation tendency. In both cases, the electron energy was also increased, leading to the larger electron beam current density and deeper penetration depth.^35,55,56^ Under these circumstances, the Eu^3+^ ions could be easily excited, finally resulting in the stronger emission intensity. In addition, based on the detected CL spectrum, the CIE coordinate was found to be (0.593, 0.399) which was located in the edge of the red region. Thus, it is noticeable that the resultant phosphor could be applied not only in WLED applications but also in FED systems.

## 4. Conclusions

The double-perovskite Gd_2−2*x*_ZnTiO_6_:2*x*Eu^3+^ phosphors with high single crystallinity were successfully synthesized *via* the solid-state reaction technique. Under the irradiation of 270 nm, the vivid red colors were emitted due to the 4f–4f transitions of Eu^3+^ ions. Since the intensity of ^5^D_0_ → ^7^F_2_ transition was the highest, it is recognizable that the Eu^3+^ ions occupied the crystallographic sites with non-inversion symmetry in the Gd_2_ZnTiO_6_ host lattice. Beyond the optimal doping concentration at *x* = 0.07, the concentration quenching occurred due to the multipole interaction. Furthermore, the Eu^3+^-activated Gd_2_ZnTiO_6_ phosphors did not only exhibited good color coordinate but also possessed high color purity of 96.8%. The studied samples possessed good thermal stability with high Δ*E* value of 0.233 eV. With the help of the prepared samples, a WLED device was prepared and it can emit bright white light with the high CRI value of 82.9. Additionally, the powders also revealed the excellent CL properties under various circumstances. These results suggest that the Gd_2−2*x*_ZnTiO_6_:2*x*Eu^3+^ phosphors can be served as promising candidates in both WLED and FED applications.

## Conflicts of interest

There are no conflicts to declare.

## Supplementary Material

RA-008-C8RA00700D-s001

## References

[cit1] Koike M., Shibata N., Kato H., Takahashi Y. (2002). IEEE J. Sel. Top. Quantum Electron..

[cit2] Steranka F. M., Bhat J., Collins D., Cook L., Craford M. G., Fletcher R., Gardner N., Grillot P., Goetz W., Keuper M., Khare R., Kim A., Krames M., Harbers G., Ludowise M., Martin P. S., Misra M., Mueller G., Mueller-Mach R., Rudaz S., Shen Y.-C., Steigerwald D., Stockman S., Subramanya S., Trottier T., Wierer J. J. (2002). Phys. Status Solidi A.

[cit3] Yang C.-Y., Som S., Das S., Lu C.-H. (2017). Sci. Rep..

[cit4] Hussain S. K., Yu J. S. (2017). RSC Adv..

[cit5] Peng M., Yin X., Tanner P. A., Brik M. G., Li P. (2015). Chem. Mater..

[cit6] Li L., Shen J., Pan Y., Zhou X., Huang H., Chang W., He Q., Wei X. (2016). Mater. Res. Bull..

[cit7] Dong G., Hou C., Yang Z., Liu P., Wang C., Lu F., Li X. (2014). Ceram. Int..

[cit8] Chiang C.-H., Fang Y.-C., Lin H.-Y., Chu S.-Y. (2017). Ceram. Int..

[cit9] Dai P., Lee S.-P., Chan T.-S., Huang C.-H., Chiang Y.-W., Chen T.-M. (2016). J. Mater. Chem. C.

[cit10] Dalal M., Taxak V. B., Chahar S., Khatkar A., Khatkar S. P. (2016). J. Phys. Chem. Solids.

[cit11] Lee S. H., Du P., Bharat L. K., Yu J. S. (2017). Ceram. Int..

[cit12] Zeng Y., Qiu K., Yang Z., Bu Y., Zhang W., Lia J. (2017). Ceram. Int..

[cit13] Chen Y., Wu K., He J., Tang Z., Shi J., Xu Y., Liu Z.-Q. (2017). J. Mater. Chem. C.

[cit14] Tang Z.-B., Xu C.-L., Wei X.-R., Zhang X.-G., Chen Y.-B. (2017). J. Alloys Compd..

[cit15] Du P., Yu J. S. (2017). Sci. Rep..

[cit16] Du P., Yu J. S. (2017). Chem. Eng. J..

[cit17] Zhang N., Zheng J., Gao J., Wu Y., Zhang R., Li T., Guo C. (2017). Dyes Pigm..

[cit18] Huang S., Li J., Wang X., Zhu Q., Sun X. (2016). Chem. Eng. J..

[cit19] Li K., Liang S., Lian H., Shang M., Xing B., Lin J. (2016). J. Mater. Chem. C.

[cit20] Liu G., Fu Z., Sheng T., Sun Z., Zhang X., Wei Y., Ma L., Wang X., Wu Z. (2016). RSC Adv..

[cit21] Guo Y., Moon B. K., Choi B. C., Jeong J. H., Kim J. H. (2017). Mater. Res. Bull..

[cit22] Liu Q., Wang L., Huang W., Li X., Yu M., Zhang Q. (2018). Ceram. Int..

[cit23] Chen P., Yang D., Hu W., Zhang J., Wu Y. (2017). Chem. Phys. Lett..

[cit24] Ding N., Zhang L., Liu Q., Xu T., Zhang Q. (2017). J. Mater. Sci..

[cit25] Yu R., Wang C., Chen J., Wu Y., Li H., Ma H. (2014). ECS J. Solid State Sci. Technol..

[cit26] Das N., Nath M. A., Thakur G. S., Thirumal M., Ganguli A. K. (2015). J. Solid State Chem..

[cit27] Chen H., Lin H., Huang Q., Huang F., Xu J., Wang B., Lin Z., Zhou J., Wang Y. (2016). J. Mater. Chem. C.

[cit28] Liao J., Wang Q., Wen H.-R., Yuan H., Liu S.-J., Fu J., Qiu B. (2017). J. Mater. Chem. C.

[cit29] Du P., Huang X., Yu J. S. (2018). Chem. Eng. J..

[cit30] Zhou J., Xia Z., Bettinelli M., Liu Q. (2016). RSC Adv..

[cit31] Zhong J., Chen D., Zhou Y., Wan Z., Ding M., Bai W., Ji Z. (2016). Dalton Trans..

[cit32] Xiao N., Shen J., Xiao T., Wu B., Luo X., Li L., Wang Z., Zhou X. (2015). Mater. Res. Bull..

[cit33] Deng T. T., Song E. H., Zhou Y. Y., Wang L. Y., Zhang Q. Y. (2017). J. Mater. Chem. C.

[cit34] Phatak R., Gupta S. K., Krishnan K., Sali S. K., Godbole S. V., Das A. (2014). Dalton Trans..

[cit35] Du P., Yu J. S. (2015). RSC Adv..

[cit36] Deng H., Gao Z., Xue N., Jeong J. H., Yu R. (2017). J. Lumin..

[cit37] Zhao J., Guo C., Li T., Su X., Zhang N., Chen J. (2016). Dyes Pigm..

[cit38] Yanga R.-Y., Peng Y.-M., Lai H.-L., Su Y.-K., Chang S.-J. (2017). Ceram. Int..

[cit39] Liu Q., Li X., Zhang B., Wang L., Zhang Q., Zhang L. (2016). Ceram. Int..

[cit40] Meza O., Villabona-Leal E. G., Diaz-Torres L. A., Desirena H., Rodríguez-López J. L., Pérez E. (2014). J. Phys. Chem. A.

[cit41] Zhang J., Yang Y., Liu Y., Mi C., Li G., Han B., Zhang Y., Seo H. J. (2015). J. Am. Ceram. Soc..

[cit42] Xin M., Tu D., Zhu H., Luo W., Liu Z., Huang P., Li R., Cao Y., Chen X. (2015). J. Mater. Chem. C.

[cit43] Jia Y., Pang R., Li H., Sun W., Fu J., Jiang L., Zhang S., Su Q., Li C., Liu R.-S. (2015). Dalton Trans..

[cit44] Blasse G. (1986). J. Solid State Chem..

[cit45] Blasse G., Bril A. (1969). J. Chem. Phys..

[cit46] Zhao C., Xia Z., Yu S. (2014). J. Mater. Chem. C.

[cit47] Du P., Yu J. S. (2016). J. Lumin..

[cit48] Wang C., Jin Y., Lv Y., Ju G., Chen L., Li Z., Hu Y. (2017). J. Lumin..

[cit49] Huang X., Guo H., Li B. (2017). J. Alloys Compd..

[cit50] Bharat L. K., Dugasani S. R., Raju G. S. R., Yu J. S. (2017). Nanotechnology.

[cit51] Wood D. L., Tauc J. (1972). Phys. Rev. B.

[cit52] Som S., Kunti A. K., Kumar V., Kumar V., Dutta S., Chowdhury M., Sharma S. K., Terblans J. J., Swart H. C. (2014). J. Appl. Phys..

[cit53] Du P., Guo Y., Lee S. H., Yu J. S. (2017). RSC Adv..

[cit54] Huang X., Li B., Guo H., Chen D. (2017). Dyes Pigm..

[cit55] Long Q., Wang C., Li Y., Ding J., Wang Y. (2016). J. Alloys Compd..

[cit56] Li G., Wang Y., Zeng W., Chen W., Han S., Guo H., Li Y. (2016). J. Mater. Chem. C.

