# Supplementary material for: Luminescent properties of Eu3+-activated Gd2ZnTiO6 double perovskite red-emitting phosphors for white light-emitting diodes and field emission displays
Source: RSC Adv. 2018 Mar 21;8(20):11207–15. doi: 10.1039/c8ra00700d (PMC9078974; doi:10.1039/c8ra00700d)
Supplement: RA-008-C8RA00700D-s001 [file RA-008-C8RA00700D-s001.pdf]

Electronic Supplementary Material (ESI) for RSC Advances.
This journal is © The Royal Society of Chemistry 2018

Luminescent properties of Eu^{3+}-activated Gd_2ZnTiO_6 double perovskite red-emitting phosphors for white light-emitting diodes and field emission displays

Soo Hyun Lee, Youngjin Cha, Hyosung Kim, Seungmoo Lee, and Jae Su Yu*

Department of Electronic Engineering, Kyung Hee University, Yongin-si, Gyeonggi-do 17104, Republic of Korea

* Corresponding author:

E-mail address: jsyu@khu.ac.kr

Tel: 82 31 201 3820

Fax: 82 31 204 8115

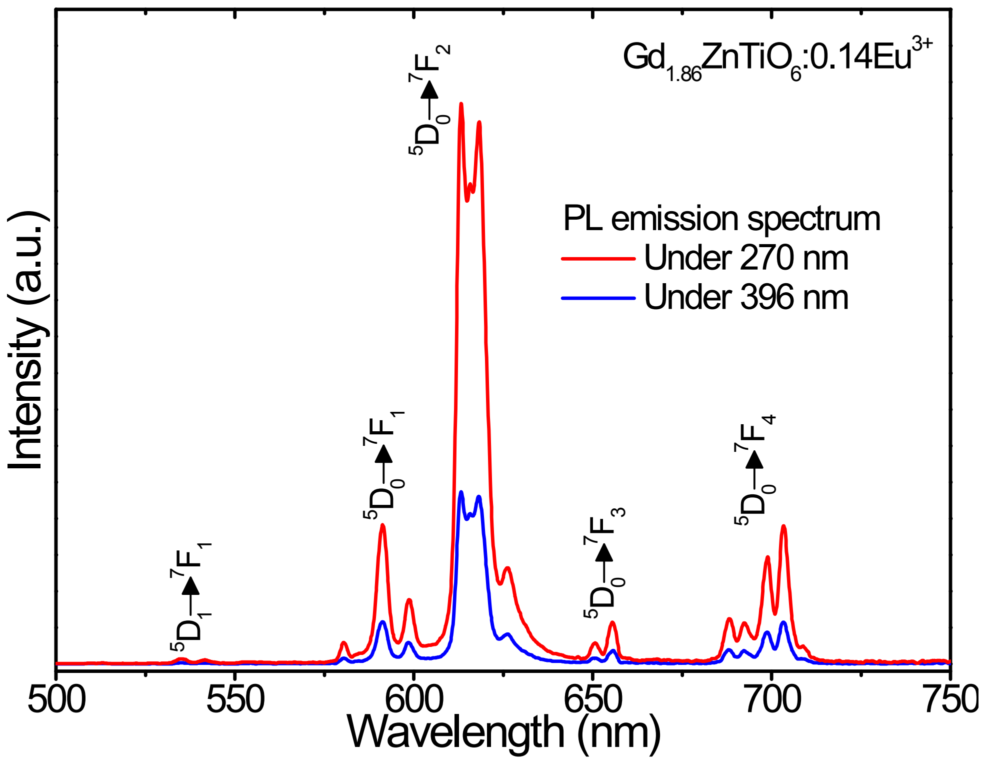

Fig. S1 PL emission spectra of the $Gd_{1.86}ZnTiO_6$:0.14Eu^{3+} phosphor under different excitation wavelengths at 270 and 396 nm.
